# Machine learning-based model for predicting recanalization in isolated distal deep vein thrombosis and analysis of predictors

**DOI:** 10.1371/journal.pone.0349110

**Published:** 2026-05-08

**Authors:** Yingjie Kuang, Jun Zhang, Zhen An, Chunxu Yang, Wenxu Guo, Xiaomin Liu, Yue Zhang

**Affiliations:** 1 Shandong University of Traditional Chinese Medicine, Jinan, China‌‌; 2 Shandong University of Traditional Chinese Medicine Affiliated Hospital, Jinan, China; 3 Xuzhou Hospital of Traditional Chinese Medicine, Jiangsu, China‌‌; Chongqing Medical University, CHINA

## Abstract

**Background:**

Isolated distal deep vein thrombosis (IDDVT) is common, yet tools for predicting poor recanalization remain limited. We aimed to develop and compare machine learning models for predicting poor recanalization in patients with IDDVT and to identify the most informative predictors.

**Methods:**

A total of 1600 patients with IDDVT were retrospectively enrolled. The dataset was randomly divided into a development set (n = 1280) and an independent test set (n = 320) using stratified sampling. Six predictive models were developed and compared: logistic regression (LR), support vector machine (SVM), random forest (RF), multilayer perceptron (MLP), extreme gradient boosting (XGBoost), and a Voting Ensemble. Model training and hyperparameter tuning were performed in the development set using five-fold stratified cross-validation, and optimal classification thresholds were determined using the Youden index. Model performance was evaluated by discrimination, calibration, and classification metrics, with 95% confidence intervals estimated by bootstrap resampling (10,000 iterations). SHAP analysis was applied to interpret the final model.

**Results:**

In the independent test set, all models showed acceptable to strong discrimination, with AUC values ranging from 0.808 to 0.908. XGBoost achieved the best overall performance, with an optimal threshold of 0.183, an AUC of 0.908 (95% CI, 0.855–0.952), a Brier score of 0.077 (95% CI, 0.058–0.096), an accuracy of 0.900 (95% CI, 0.866–0.931), a precision of 0.650 (95% CI, 0.529–0.767), a recall of 0.803 (95% CI, 0.686–0.906), an F1-score of 0.717 (95% CI, 0.615–0.806), and a specificity of 0.918 (95% CI, 0.884–0.950). The calibration intercept and slope of the XGBoost model were 0.149 (95% CI, −0.192 to 0.454) and 1.410 (95% CI, 1.098–1.809), respectively, indicating acceptable overall calibration. SHAP analysis identified D-dimer rate, provoking-factor-related variables, anticoagulant use, and age group as the most influential predictors.

**Conclusion:**

Among six candidate models, XGBoost showed the best overall performance for predicting poor recanalization in patients with IDDVT. This study establishes an interpretable machine learning-based prediction framework focused specifically on poor recanalization in IDDVT and highlights the contribution of dynamic laboratory information, particularly D-dimer rate. The model may support early risk stratification and individualized follow-up planning, but external validation is required before routine clinical implementation.

## Introduction

Isolated distal deep vein thrombosis (IDDVT), a subtype of deep vein thrombosis (DVT), is defined as thrombosis occurring in the infra-popliteal veins, including the anterior tibial, posterior tibial, peroneal veins, and the muscular venous plexus [1]. Following the onset of venous thrombosis, the successful restoration of vascular recanalization and the reduction of residual vein occlusion are critical factors for clinical recovery. Prompt recanalization can effectively reduce the risk of thrombus recurrence, pulmonary embolism (PE), and post-thrombotic syndrome (PTS) [2]. Given that the risk profile of IDDVT differs from that of proximal deep vein thrombosis (PDVT), IDDVT is more strongly associated with transient provoking factors, such as recent surgery, immobilization of the affected limb, or long-distance travel, whereas PDVT is more closely linked to persistent provoking factors, including active cancer, congestive heart failure, respiratory insufficiency, and advanced age (>75 years) [3,4]. Therefore, findings derived from PDVT studies cannot be directly extrapolated to IDDVT. Previous studies have shown that the incidence of PTS after IDDVT may be as high as 17%, and poor venous recanalization after thrombosis is strongly associated with PTS [5]. Accordingly, studies specifically aimed at evaluating recanalization outcomes in IDDVT are warranted.

Clinical prediction models are widely used for the prevention and management of DVT. These models assess DVT risk factors and play a crucial role in reducing the incidence of initial thrombosis, preventing recurrence, and mitigating PTS [6–8]. The Wells score is the most widely used clinical prediction tool and was originally developed to estimate the probability of PE and DVT; however, its diagnostic accuracy for IDDVT is relatively limited [9]. Therefore, it is not well suited for assessing the risk of IDDVT. Existing prediction studies focusing on IDDVT have mainly addressed the occurrence of IDDVT itself or the development of pulmonary embolism, whereas relatively few studies have used venous recanalization in IDDVT as the primary endpoint [10–12]. Compared with traditional multivariable regression, machine learning methods may be better suited to clinical prediction tasks involving complex nonlinear associations, higher-order interactions, and heterogeneous predictors. In IDDVT, where recanalization is likely influenced by multiple interrelated clinical and laboratory factors, machine learning may provide complementary value for individualized risk prediction.

Accordingly, this study aimed to retrospectively analyze patient data to develop a machine learning-based model for predicting poor recanalization in IDDVT, identify the most informative predictors, and provide supportive evidence for clinical decision-making.

## Methods

### Study dataset construction

#### Data source and exclusion criteria.

All data in this study were obtained from the Affiliated Hospital of Shandong University of Traditional Chinese Medicine. The study cohort comprised patients diagnosed with IDDVT between January 1, 2020 and October 31, 2023. Exclusion criteria comprised: (1) concomitant venous thrombosis at other sites; (2) interrupted treatment courses; and (3) incomplete medical documentation.

#### Sample size determination.

This study was a retrospective clinical prediction model development study using machine learning algorithms. Sample size considerations were informed by published methodological guidance for clinical prediction modeling and by the TRIPOD+AI reporting recommendations [13]. Based on the anticipated poor recanalization rate, the number of candidate predictors, and the planned model complexity, the available sample size was considered adequate for model development. Based on the preliminary poor recanalization rate (14.8%), 20 candidate predictors, and the expected model discrimination, the minimum required sample size for model development was estimated to be approximately 679 patients. Given the greater complexity of the machine learning algorithms planned in this study, the actual sample size requirement is generally higher than the minimum required for traditional regression models. Accordingly, all 1,600 eligible patients with IDDVT were included in the final analysis.

### Study design

**Fig 1:**
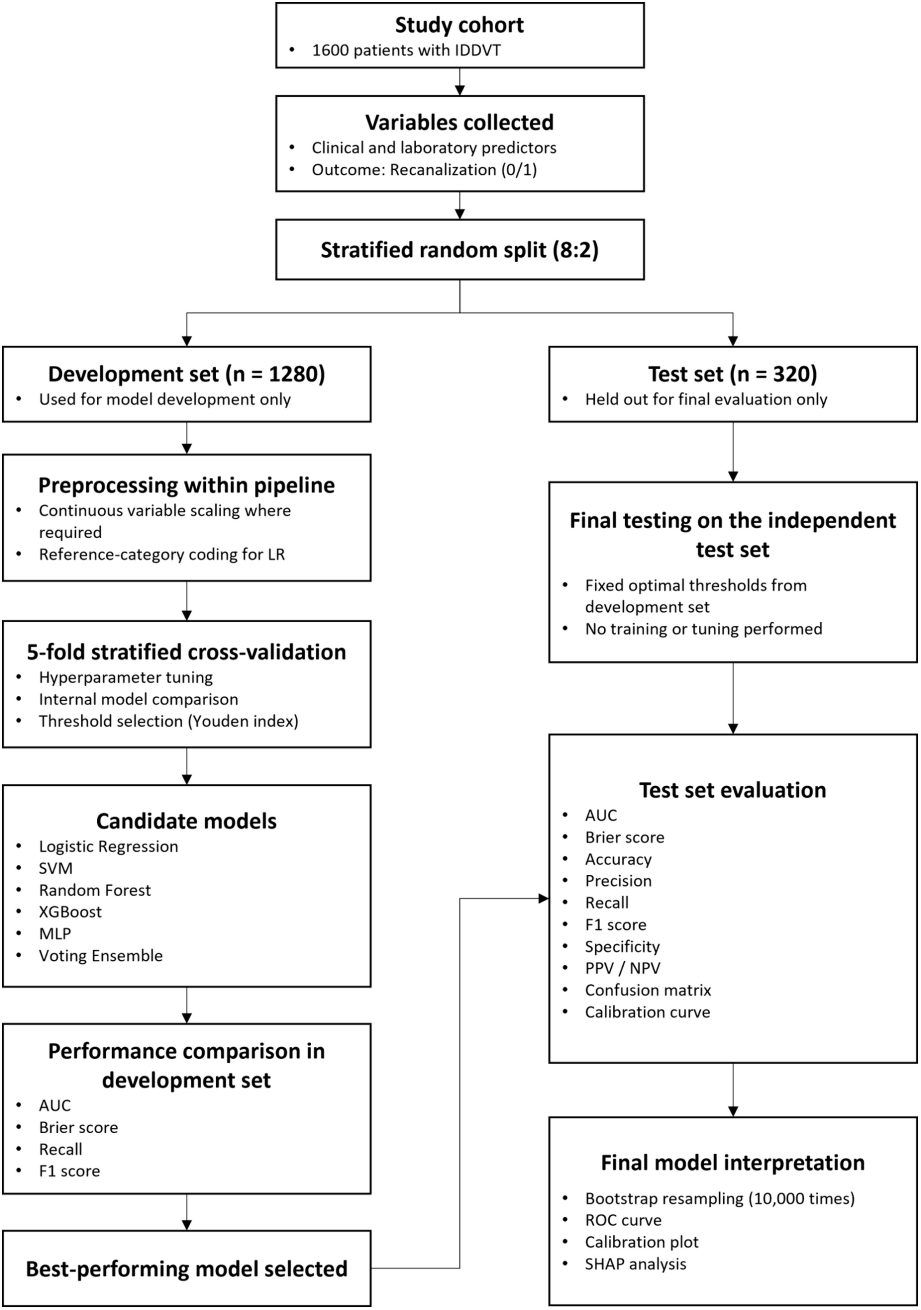
Workflow of machine learning model development, selection, and final evaluation.

The overall workflow of data splitting, model development, selection, and final evaluation is illustrated in Fig 1.

#### Group stratification.

Venous recanalization rates were determined using lower extremity venous Doppler ultrasound reports. Patients with a recanalization rate of <50% were assigned to the poor recanalization group, whereas those with a recanalization rate of ≥50% were classified into the good recanalization group. The detailed methodology for calculating the recanalization rate is provided in S1 File. The poor recanalization group was coded as 1 and the good recanalization group as 0. For comparisons of baseline characteristics between groups, categorical variables were presented as counts (percentages) and analyzed using the chi-square test, with Fisher’s exact test applied when appropriate. Continuous variables were summarized and compared according to their distributional characteristics: variables with approximately normal distributions were presented as mean ± standard deviation and compared using Welch’s t-test, whereas non-normally distributed variables were presented as median (interquartile range) and compared using the Mann–Whitney U test. All tests were two-sided, and a P value < 0.05 was considered statistically significant. Patients in each group (poor recanalization and good recanalization) were randomly allocated to the development and test sets in an 80:20 ratio, while preserving the class distribution within each group.

#### Candidate predictors.

Candidate predictors were selected based on the published literature, clinical experience, and research team consensus, including sex, age, body mass index (BMI), thrombus location (left-sided, right-sided, or bilateral), outpatient/inpatient status, family history of VTE, and personal history of VTE. Additional factors included provoking factor type, international normalized ratio (INR), platelet count (PLT), fibrinogen (FIB), D-dimer, C-reactive protein (CRP), the rate of change in D-dimer levels, and anticoagulant therapy use. In our center, anticoagulant therapy for patients with IDDVT mainly consisted of subcutaneous low-molecular-weight heparin and oral rivaroxaban. In total, 20 candidate predictors were included in the analysis. Detailed calculation methods for some included predictors are provided in S2 File.

#### Data preprocessing.

Because several variables had missing values and the available complete-case sample remained sufficiently large for model development, a complete-case analysis was performed. Continuous variables were standardized to improve comparability across different measurement scales and value ranges, with all preprocessing performed within the model pipeline to avoid data leakage. Specifically, preprocessing parameters were fitted only on the training data during model development and then applied to the corresponding validation and test data. Categorical variables were one-hot encoded. Collinearity diagnostics were performed for all candidate predictors, with detailed results provided in S3 File. The results showed strong correlations among some variables, mainly among mutually exclusive dummy variables derived from the same multicategory variable, such as those representing provoking factor type and lesion location. This finding primarily reflects the structural characteristics of one-hot encoding rather than abnormal overlap among independent clinical factors. Accordingly, reference-category coding was used in the logistic regression model to reduce the influence of multicollinearity on parameter estimation and interpretation. Specifically, transient provoking factors were specified as the reference category for provoking factor type, and left-sided location as the reference category for lesion location, while the remaining categories were included as dummy variables.

#### Predictive model construction.

To compare algorithms with different modeling characteristics and levels of complexity, six predictive models were selected in this study. Logistic regression (LR) was included as a conventional and interpretable baseline model; support vector machine (SVM) was used because of its ability to handle complex decision boundaries; random forest (RF) and extreme gradient boosting (XGBoost) were included as tree-based ensemble methods capable of capturing nonlinear relationships and feature interactions; multilayer perceptron (MLP) was used as a neural-network-based approach; and a Voting Ensemble was included to examine whether combining multiple models could provide complementary predictive value. Based on these considerations, six machine learning algorithms were applied, including LR, XGBoost, RF, MLP, SVM, and a Voting Ensemble. Within the development set, five-fold stratified cross-validation was used for hyperparameter tuning and internal performance assessment. The independent test set was reserved exclusively for final model evaluation. For each model, hyperparameters were optimized within the development set, and the classification threshold was determined using the Youden index and then fixed for evaluation in the independent test set. Model performance was subsequently assessed in the test set.

#### Model performance evaluation.

Model performance was assessed using sensitivity, specificity, F1-score, accuracy, the area under the receiver operating characteristic curve (AUC), and the Brier score. Discrimination was primarily assessed using the AUC, with higher values indicating better ability to distinguish between patients with and without poor recanalization [14]. SHapley Additive exPlanations (SHAP) analysis was used to visualize feature importance and direction of association through SHAP summary plots. For the final selected model, SHAP values were computed using the independent test set after applying the preprocessing pipeline fitted during model development. This method improved model interpretability and provided intuitive explanations for individual predictions.

### Ethics

This retrospective analysis utilized fully de-identified data, containing no personally identifiable information. The study protocol was approved by the Institutional Review Board of the Affiliated Hospital of Shandong University of Traditional Chinese Medicine (Approval No. 2024021-YJS). The requirement for informed consent was waived by the ethics committee due to the retrospective nature of the study and use of anonymized data.

### Model development environment

All analyses were performed in a Jupyter Notebook environment using Python 3.11.5 for data processing, model development, and statistical analysis. The main Python packages used were pandas (2.1.4), numpy (1.24.3), scikit-learn (1.1.3), xgboost (1.7.3), shap (0.46.0), matplotlib (3.7.2), scipy (1.15.3), and statsmodels (0.14.6).

## Results

### Baseline characteristics of the study cohort

**Fig 2:**
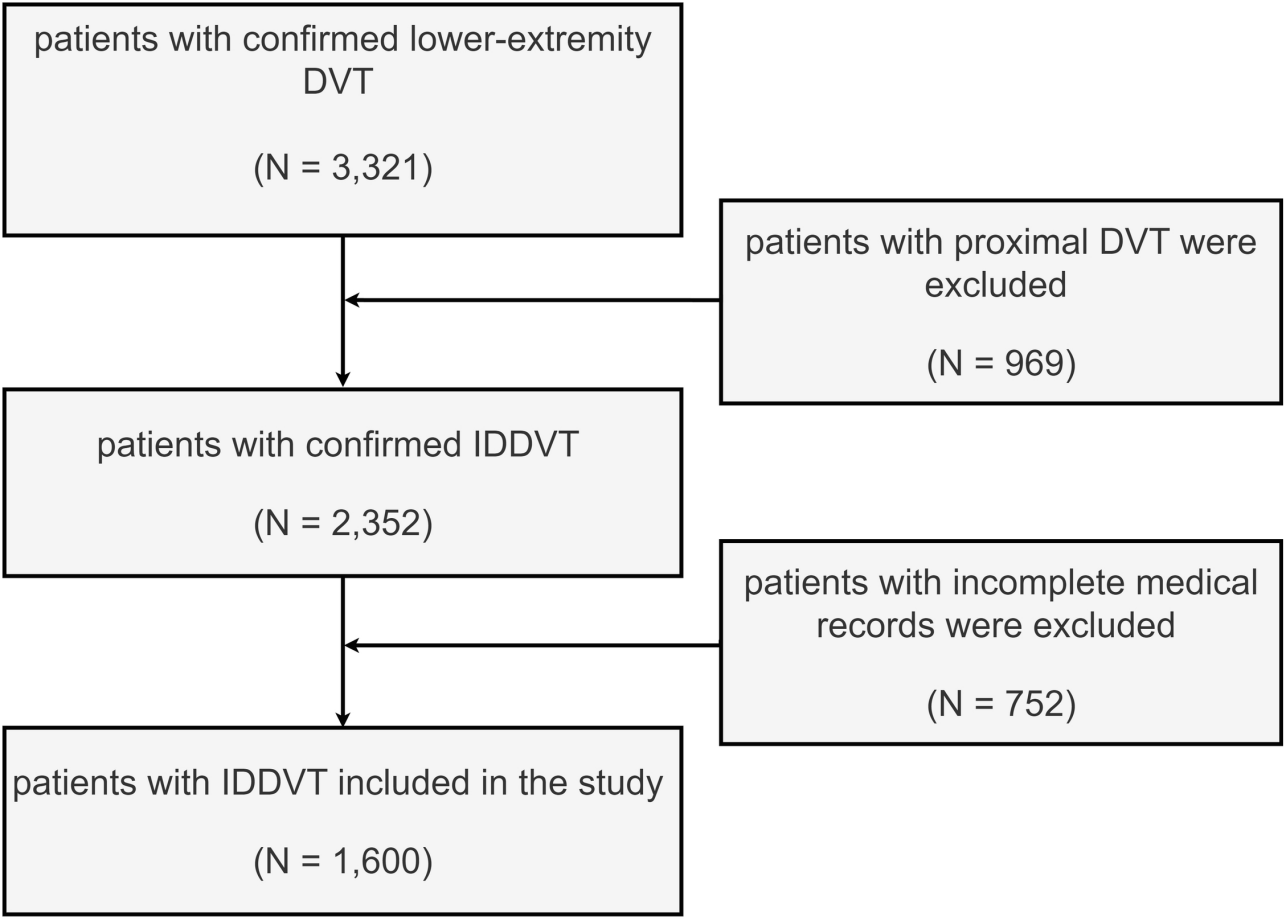
Flowchart of patient selection.

A total of 2,352 IDDVT patient records were initially screened for this study, among which 752 cases were excluded due to missing data. Ultimately, 1,600 IDDVT patients were included in the final analysis (Fig 2). Of these, 1,347 patients (84.2%) achieved good recanalization at the 1-month follow-up, while 253 patients (15.8%) showed poor recanalization outcomes. The baseline characteristics of the included patients are summarized in Table 1. The baseline characteristics were generally comparable between the development and test sets, with no marked imbalance observed, indicating that the random stratified split was reasonable (S1 Table).

**Table 1:** Baseline characteristics according to recanalization outcome.

Variable	Category	Overall	Good Recanalization Group	Poor Recanalization Group	*P* value
Sex	Male	796 (49.8%)	670 (49.7%)	126 (49.8%)	1.000
	Female	804 (50.2%)	677 (50.3%)	127 (50.2%)	
Age	> 60 years	933 (58.3%)	761 (56.5%)	172 (68.0%)	<0.001
	≤ 60 years	667 (41.7%)	586 (43.5%)	81 (32.0%)	
Thrombus location	Bilateral	160 (10.0%)	129 (9.6%)	31 (12.3%)	0.420
	Left side	729 (45.6%)	618 (45.9%)	111 (43.9%)	
	Right side	711 (44.4%)	600 (44.5%)	111 (43.9%)	
Hospitalization status	Inpatient	1037 (64.8%)	866 (64.3%)	171 (67.6%)	0.349
	Outpatient	563 (35.2%)	481 (35.7%)	82 (32.4%)	
Family history	Yes	73 (4.6%)	56 (4.2%)	17 (6.7%)	0.104
	No	1527 (95.4%)	1291 (95.8%)	236 (93.3%)	
Provoking factors	Transient provoking factors	1157 (72.3%)	1088 (80.8%)	69 (27.3%)	<0.001
	Persistent provoking factors	266 (16.6%)	127 (9.4%)	139 (54.9%)	
	Multiple provoking factors	74 (4.6%)	35 (2.6%)	39 (15.4%)	
	Unprovoked	103 (6.4%)	97 (7.2%)	6 (2.4%)	
VTE history	Recurrent VTE	261 (16.3%)	207 (15.4%)	54 (21.3%)	0.023
	First episode	1339 (83.7%)	1140 (84.6%)	199 (78.7%)	
Anticoagulant therapy	Yes	1127 (70.4%)	982 (72.9%)	145 (57.3%)	<0.001
	No	473 (29.6%)	365 (27.1%)	108 (42.7%)	
Laboratory parameters	BMI, kg/m²	23.91 ± 3.26	23.87 ± 3.28	24.10 ± 3.16	0.308
	CRP, mg/L	5.06 (2.74, 7.30)	4.99 (2.68, 7.36)	5.27 (2.78, 7.16)	0.663
	Platelet count, × 10⁹/L	235.00 (174.00, 292.00)	235.00 (175.00, 292.00)	228.00 (172.00, 292.00)	0.698
	INR	1.06 (0.97, 1.15)	1.06 (0.97, 1.15)	1.06 (0.97, 1.14)	0.761
	Fibrinogen, g/L	3.09 (2.53, 3.65)	3.07 (2.53, 3.65)	3.18 (2.52, 3.68)	0.516
	D-dimer, μg/mL	1.98 (1.25, 2.67)	1.94 (1.23, 2.65)	2.20 (1.33, 2.78)	0.077
	D-dimer rate, %	0.25 (0.09, 0.42)	0.28 (0.12, 0.44)	0.13 (−0.01, 0.29)	<0.001

### Model development

This study developed and evaluated six prediction models, including LR, SVM, RF, MLP, XGBoost, and a Voting Ensemble. All models were trained and hyperparameter-optimized in the development set using five-fold stratified cross-validation, and the optimal classification threshold for each model was determined according to the Youden index. Because poor recanalization events were relatively infrequent in the study cohort, classification thresholds were determined within the development set to achieve an appropriate balance between sensitivity and specificity before being fixed for evaluation in the independent test set.

**Fig 3:**
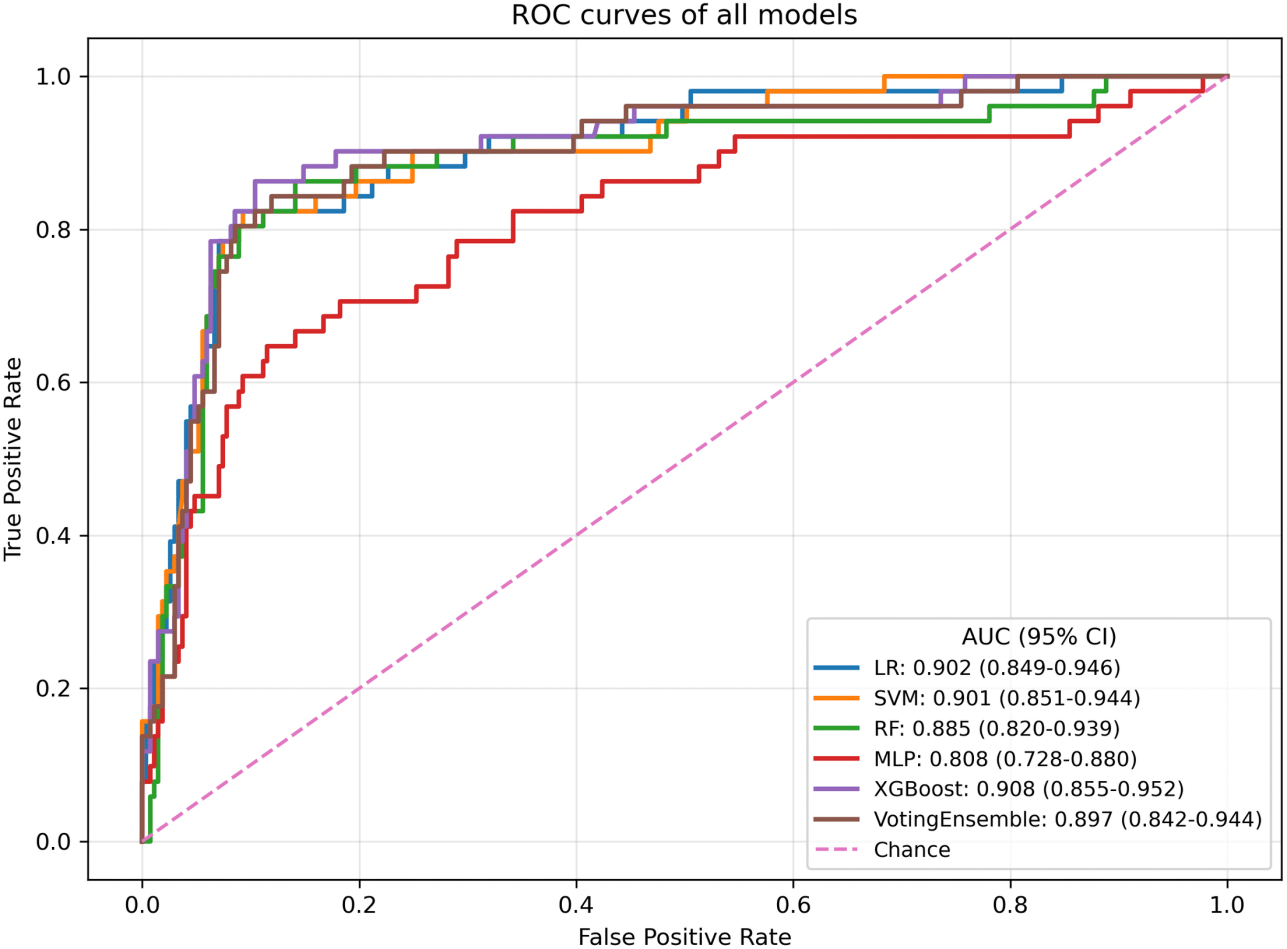
Receiver operating characteristic curves of the candidate models.

In the independent test set, the AUCs of the models ranged from 0.808 to 0.908, indicating overall good discrimination (Fig 3). XGBoost achieved the best performance, with an optimal threshold of 0.183, an AUC of 0.908 (95% CI: 0.855–0.952), a Brier score of 0.077 (95% CI: 0.058–0.096), an accuracy of 0.900 (95% CI: 0.866–0.931), a precision of 0.650 (95% CI: 0.529–0.767), a recall of 0.803 (95% CI: 0.686–0.906), an F1-score of 0.717 (95% CI: 0.615–0.806), and a specificity of 0.918 (95% CI: 0.884–0.950). SVM and LR also performed favorably; SVM showed better balance in classification performance, with an F1-score of 0.718 (95% CI: 0.614–0.808), whereas LR achieved a higher recall of 0.822 (95% CI: 0.712–0.922). RF and the Voting Ensemble showed intermediate performance, whereas MLP demonstrated relatively weaker overall performance (Table 2). The detailed classification results are further illustrated by the confusion matrices of all candidate models in the development and independent test sets, which are provided in S1 and S2 Figs, respectively.

**Table 2:** Performance of machine learning models for predicting poor recanalization in the test set.

Model	AUC	Brier	Accuracy	Precision	Recall	F1	Specificity	NPV	Threshold
LR	0.902 (0.849–0.946)	0.081 (0.061–0.102)	0.841 (0.800–0.878)	0.499 (0.394–0.610)	0.822 (0.712–0.922)	0.620 (0.520–0.712)	0.844 (0.799–0.887)	0.962 (0.936–0.983)	0.140
SVM	0.901 (0.851–0.944)	0.082 (0.063–0.103)	0.903 (0.869–0.934)	0.666 (0.542–0.783)	0.783 (0.667–0.892)	0.718 (0.614–0.808)	0.926 (0.893–0.956)	0.958 (0.931–0.981)	0.220
RF	0.885 (0.820–0.939)	0.085 (0.067–0.104)	0.900 (0.866–0.931)	0.686 (0.556–0.812)	0.685 (0.552–0.809)	0.683 (0.571–0.785)	0.941 (0.911–0.967)	0.940 (0.911–0.967)	0.432
MLP	0.808 (0.728–0.880)	0.117 (0.086–0.149)	0.872 (0.834–0.906)	0.646 (0.484–0.806)	0.430 (0.293–0.571)	0.513 (0.375–0.640)	0.955 (0.930–0.978)	0.899 (0.863–0.932)	0.752
XGBoost	0.908 (0.855–0.952)	0.077 (0.058–0.096)	0.900 (0.866–0.931)	0.650 (0.529–0.767)	0.803 (0.686–0.906)	0.717 (0.615–0.806)	0.918 (0.884–0.950)	0.961 (0.936–0.983)	0.183
Voting Ensemble	0.897 (0.842–0.944)	0.082 (0.063–0.104)	0.888 (0.850–0.922)	0.647 (0.511–0.780)	0.646 (0.509–0.774)	0.644 (0.529–0.750)	0.933 (0.902–0.962)	0.933 (0.901–0.960)	0.352

Data are presented as estimates (95% CI). Abbreviations: NPV, negative predictive value.

**Fig 4:**
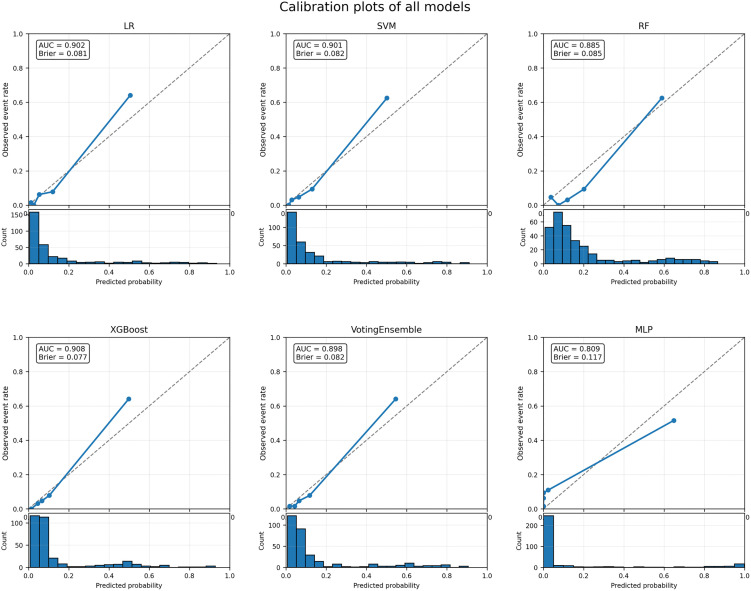
Calibration curves of the prediction models.

Calibration curve analysis showed that the predicted probabilities of the compared models were generally in good agreement with the observed event rates, although some differences were noted across models (Fig 4). Together with its lower Brier score, XGBoost demonstrated favorable overall calibration performance. Further calibration assessment showed that the calibration intercept of XGBoost was 0.149 (95% CI: −0.192 to 0.454), which was close to the ideal value of 0, indicating no evident systematic overestimation or underestimation of risk. The calibration slope was 1.410 (95% CI: 1.098–1.809), which was higher than the ideal value of 1, suggesting that the predicted probabilities may have been insufficiently extreme overall. Overall, XGBoost achieved acceptable calibration while maintaining strong discriminative ability.

Overall, XGBoost demonstrated the best performance in terms of discrimination, calibration, and overall classification performance, and was therefore selected as the optimal final model.

### Analysis of important predictors

**Fig 5:**
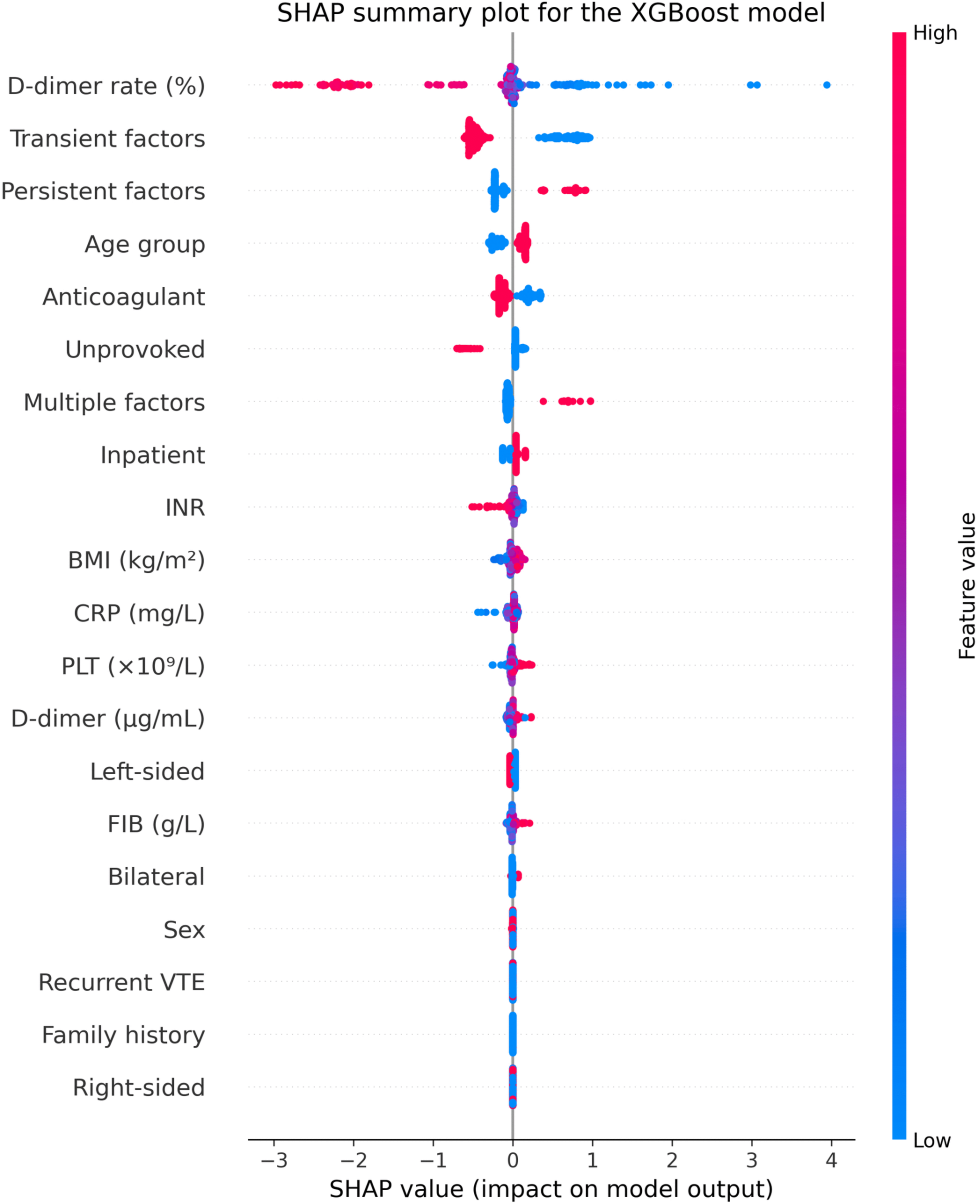
SHAP summary plot of predictor contributions in the independent test set for the final XGBoost Model.

To interpret the prediction mechanism of the best-performing XGBoost model, SHAP was further used to evaluate the contribution of each predictor to the model output in the independent test set. The SHAP summary plot (Fig 5) showed that the D-dimer rate, variables representing provoking factor type, age group, and anticoagulant therapy were the main contributors to model predictions. In our analysis, poor recanalization was coded as 1 and good recanalization as 0. Therefore, positive SHAP values indicate that a feature shifts the model output toward class 1 (poor recanalization), whereas negative SHAP values indicate a shift toward class 0 (good recanalization). Specifically, a lower D-dimer rate was associated with a greater tendency toward predicted poor recanalization; persistent provoking factors, multiple provoking factors, and older age groups tended to shift the model toward poor recanalization, whereas transient provoking factors and anticoagulant therapy tended to shift the model toward good recanalization. It should be noted that the variables representing provoking factor type were derived from one-hot encoding of the same multicategory variable. Therefore, their SHAP values reflect the relative predictive contribution of each category within the overall encoding framework and should not be directly interpreted as independent risk effects.

## Discussion

In this study, six models were developed and compared for predicting the risk of poor recanalization in patients with IDDVT, including logistic regression, support vector machine, random forest, multilayer perceptron, XGBoost, and a Voting Ensemble. Among the candidate models, XGBoost achieved the best overall performance in the independent test set. Specifically, it showed the highest discriminative ability, the lowest Brier score, and acceptable calibration, indicating a favorable balance between classification performance and probability estimation. In addition, SHAP-based interpretation enhanced the transparency of the final model by identifying the key variables driving the predictions. Taken together, these findings support the potential utility of machine learning approaches, particularly XGBoost, in predicting the risk of poor recanalization in patients with IDDVT.

SHAP analysis further enhanced the interpretability of the best-performing XGBoost model and identified the key predictors contributing most to the model output. The results showed that the D-dimer rate, variables representing provoking factor type, anticoagulant therapy, and age group were the main drivers of model predictions. These findings are clinically plausible. Dynamic changes in D-dimer may reflect persistent thrombus burden and fibrinolytic activity and may therefore provide more information relevant to recanalization than a single baseline measurement [15,16]. Variables representing provoking factor type may capture differences in the thrombotic background and the persistence of risk exposure, both of which may substantially affect prognosis [17,18]. Likewise, age and anticoagulant therapy are closely related to thrombus resolution and treatment response, which may explain their high importance in the model. In addition, the baseline statistical analysis showed that older age, recurrent VTE, provoking factor type, anticoagulant therapy, and D-dimer rate differed significantly between the good and poor recanalization groups. These findings are broadly consistent with the predictors highlighted by the final model and support the clinical relevance of these variables. In particular, a history of recurrent VTE may reflect an underlying prothrombotic tendency or a more complex disease background, which could be associated with less favorable thrombus resolution. Nevertheless, these associations should be interpreted cautiously, because the present study was designed primarily for prediction rather than for causal inference. In addition, it should be emphasized that SHAP results reflect the relative contribution of features to model predictions rather than evidence of independent causal effects. Because some overlap was observed in the SHAP distributions of these variables, their roles should be interpreted as joint contributions to model prediction behavior within the overall encoding framework, rather than as mutually independent causal effects. Accordingly, the principal value of SHAP analysis lies in facilitating interpretation of the model’s overall predictive logic, rather than directly establishing causal relationships between individual predictors and recanalization outcomes.

Another important issue is the feasibility of implementing the model in real-world clinical settings. In the final model, the D-dimer rate emerged as one of the most important predictors, suggesting that serial changes in coagulation-related biomarkers may carry additional prognostic information in this cohort. Previous studies have reported a positive linear association between dynamic changes in D-dimer levels and thrombus resolution rate and have suggested their potential value in predicting recanalization outcomes in patients with pulmonary embolism [19,20]. However, this variable depends on serial D-dimer measurements rather than a single baseline measurement, and serial D-dimer testing has not been uniformly incorporated into routine clinical practice. In addition, institutions may differ in sampling time points, testing frequency, and laboratory procedures, which could affect the availability and consistency of this predictor in real-world settings. Therefore, this study should be viewed as exploratory, aiming to evaluate the predictive value of the D-dimer rate in this cohort rather than to demonstrate its direct applicability across all clinical settings. Before wider implementation, multicenter external validation under different testing conditions is still required, and simplified models that do not rely on serial D-dimer-related indicators should be further explored.

From a clinical perspective, the main potential value of this model lies in enabling early risk stratification for poor recanalization in patients with IDDVT, rather than functioning as a standalone tool for treatment decision-making. By identifying patients at higher risk of poor recanalization, the model may assist in tailoring follow-up intensity and monitoring strategies. Given the clinical characteristics of IDDVT, many guidelines support anticoagulation in selected patients, although uncertainties remain regarding the optimal timing of treatment initiation and the most appropriate treatment strategy in specific clinical scenarios [21–23]. Accordingly, in clinical practice, the model developed in this study may support closer ultrasound surveillance, more careful evaluation of treatment response, or earlier specialist reassessment for high-risk patients, whereas a relatively routine follow-up strategy may be considered for those at lower risk.

Previous studies have primarily focused on proximal DVT and the occurrence of PE. As a distinct subtype of lower-extremity DVT, IDDVT has historically received less attention because of its relatively mild symptoms. However, with the growing body of research in this field and advances in diagnostic methods, IDDVT has attracted increasing attention owing to its high incidence and risk of adverse outcomes [5,24]. The present study focused on poor recanalization in IDDVT as a clinically relevant outcome, developed a risk prediction model using machine learning algorithms, and provided an interpretable modeling framework while incorporating dynamic laboratory indicators. In this respect, it may offer a useful complement to previous DVT-related research.

Several limitations of this study should be noted. First, this was a single-center retrospective study. Although an independent test set was used for validation, true external validation is still lacking. Therefore, the generalizability of the model to populations with different demographic and clinical characteristics remains to be established, including younger patients and those receiving different anticoagulation regimens. Second, as an observational study, this analysis could not completely eliminate the possibility of residual confounding. For example, although early ambulation in outpatients may facilitate thrombus recanalization [25,26], outpatient status and some treatment-related variables may also partly reflect milder disease severity, lower thrombus burden, or clinical decision-making processes, rather than direct biological effects on recanalization. Third, although SHAP analysis improved model interpretability, overlap in the SHAP distributions of some variables was observed, suggesting that feature effects at the individual level are not always fully separable. Accordingly, these findings should be interpreted as reflecting the relative contribution of features to model predictions rather than as evidence of causal relationships. In addition, the use of complete-case analysis may have introduced selection bias, because patients with missing data were excluded from model development and evaluation. Finally, although the model showed generally stable performance and a certain degree of robustness, further model simplification, multicenter external validation, and prospective evaluation are still required before wider clinical implementation. Future studies should focus on assessing model performance across heterogeneous clinical workflows and on exploring simplified or dynamically updated prediction models to improve real-world applicability.

## Conclusion

Among six candidate models, XGBoost showed the best overall performance for predicting poor recanalization in patients with IDDVT, with strong discrimination (AUC 0.908), low prediction error (Brier score 0.077), acceptable calibration, and good interpretability through SHAP analysis. This study establishes an interpretable machine learning framework specifically for poor recanalization risk prediction in IDDVT and highlights the predictive contribution of dynamic laboratory information, particularly D-dimer rate. The model may support early risk stratification and individualized follow-up planning, although multicenter external validation is still required before routine clinical implementation.

## Supporting information

S1 Fig Confusion matrices of all candidate models in the development set. (PNG)

S2 Fig Confusion matrices of all candidate models in the independent test set. (PNG)

S1 File Method for calculating the venous recanalization rate. (PDF)

S2 File Calculation methods for selected predictors. (PDF)

S3 File Collinearity diagnostics for all candidate predictors and for predictors included in the logistic regression model. (PDF)

S1 Table Comparison of baseline characteristics between the development and test sets. (PDF)

S2 Table List of abbreviations used in the manuscript. (PDF)
