# Supplementary figures and images for: Machine learning-based model for predicting recanalization in isolated distal deep vein thrombosis and analysis of predictors
Source: PLoS One. 2026 May 8;21(5):e0349110. doi: 10.1371/journal.pone.0349110 (PMC13155594; doi:10.1371/journal.pone.0349110)

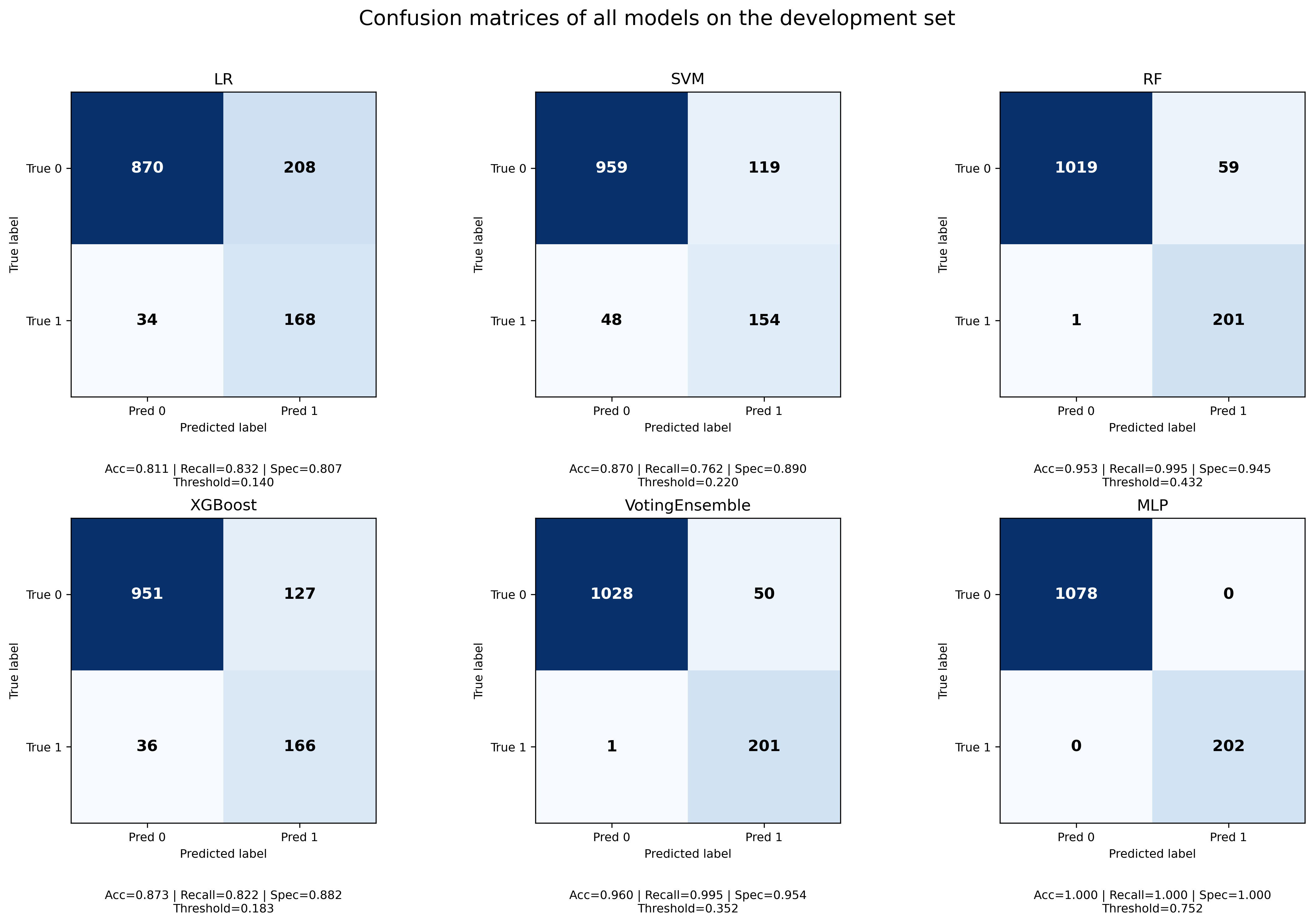

Supplement: S1 Fig — (PNG) [file pone.0349110.s001.png]

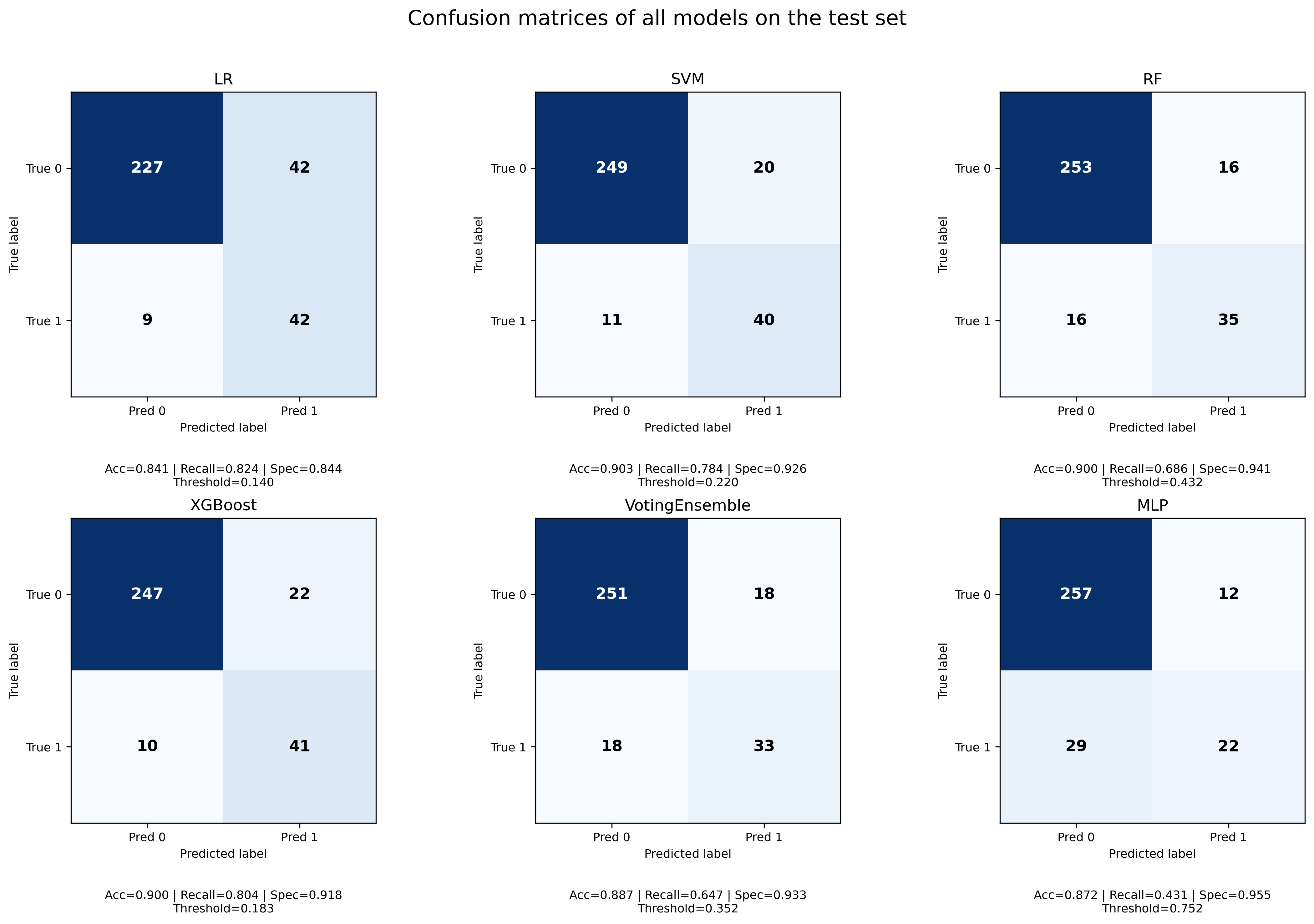

Supplement: S2 Fig — (PNG) [file pone.0349110.s002.png]
