# Supplementary material for: Machine learning-based model for predicting recanalization in isolated distal deep vein thrombosis and analysis of predictors
Source: PLoS One. 2026 May 8;21(5):e0349110. doi: 10.1371/journal.pone.0349110 (PMC13155594; doi:10.1371/journal.pone.0349110)
Supplement: S1 File — (PDF) [file pone.0349110.s003.pdf]

S1. Method for Calculating Venous Recanalization Rate

The veins affected by IDDVT are categorized as the anterior tibial vein, posterior tibial vein, peroneal vein, and muscular venous plexus. The vascular recanalization status of each affected vein is evaluated.

The criteria for lower extremity venous Doppler ultrasound are as follows:

- If the venous vessel cannot be compressed and there is no blood flow signal within the lumen, it is considered non-recanalized.
- If there is both residual thrombus and blood flow within the venous lumen, the ratio of the maximum thrombus width to the vessel diameter is calculated. A ratio of 0% to 20% is considered minimal recanalization, 20% to 40% is considered partial recanalization, 40% to 60% is considered moderate recanalization, 60% to 80% is considered substantial recanalization, and 80% to 100% is considered near-complete recanalization.
- If there is no residual thrombus and the ultrasound probe can completely compress the venous vessel to below 2 mm, it is considered complete recanalization.

The scoring system based on the findings is as follows:

- Complete or near-complete recanalization of the thrombotic vein: 0 points
- Substantial recanalization of the thrombotic vein: 1 point
- Moderate recanalization of the thrombotic vein: 2 points
- Partial recanalization of the thrombotic vein: 3 points
- Minimal recanalization or non-recanalization of the thrombotic vein: 4 points

The total score for each vein is calculated, with the same method used before and after treatment. The formula for the venous patency score is as follows:

Venous Patency Score = (Left side: anterior tibial vein + posterior tibial vein + peroneal vein + muscular venous plexus) + (Right side: anterior tibial vein + posterior tibial vein + peroneal vein + muscular venous plexus).

The venous recanalization rate at 1 month post-treatment for IDDVT patients is calculated as:

Venous Recanalization Rate = [(Pre-treatment venous patency score - Post-treatment

venous patency score) / Pre-treatment venous patency score] × 100%.

A recanalization rate ≥50% is considered good recanalization, while a rate <50% is considered poor recanalization.
