# Supplementary material for: Machine learning-based model for predicting recanalization in isolated distal deep vein thrombosis and analysis of predictors
Source: PLoS One. 2026 May 8;21(5):e0349110. doi: 10.1371/journal.pone.0349110 (PMC13155594; doi:10.1371/journal.pone.0349110)
Supplement: S2 File — (PDF) [file pone.0349110.s004.pdf]

BMI:

BMI = (Weight in kg) / (Height in m)^2

D-dimer Rate:

D-dimer Rate = ((D-dimer value on the day of diagnosis or the following day) - (D dimer value on the 7th day)) / (D-dimer value on the day of diagnosis or the following day) × 100%
