# Supplementary material for: Machine learning-based model for predicting recanalization in isolated distal deep vein thrombosis and analysis of predictors
Source: PLoS One. 2026 May 8;21(5):e0349110. doi: 10.1371/journal.pone.0349110 (PMC13155594; doi:10.1371/journal.pone.0349110)
Supplement: S3 File — (PDF) [file pone.0349110.s005.pdf]

Correlation Heatmap of All Candidate Predictors:

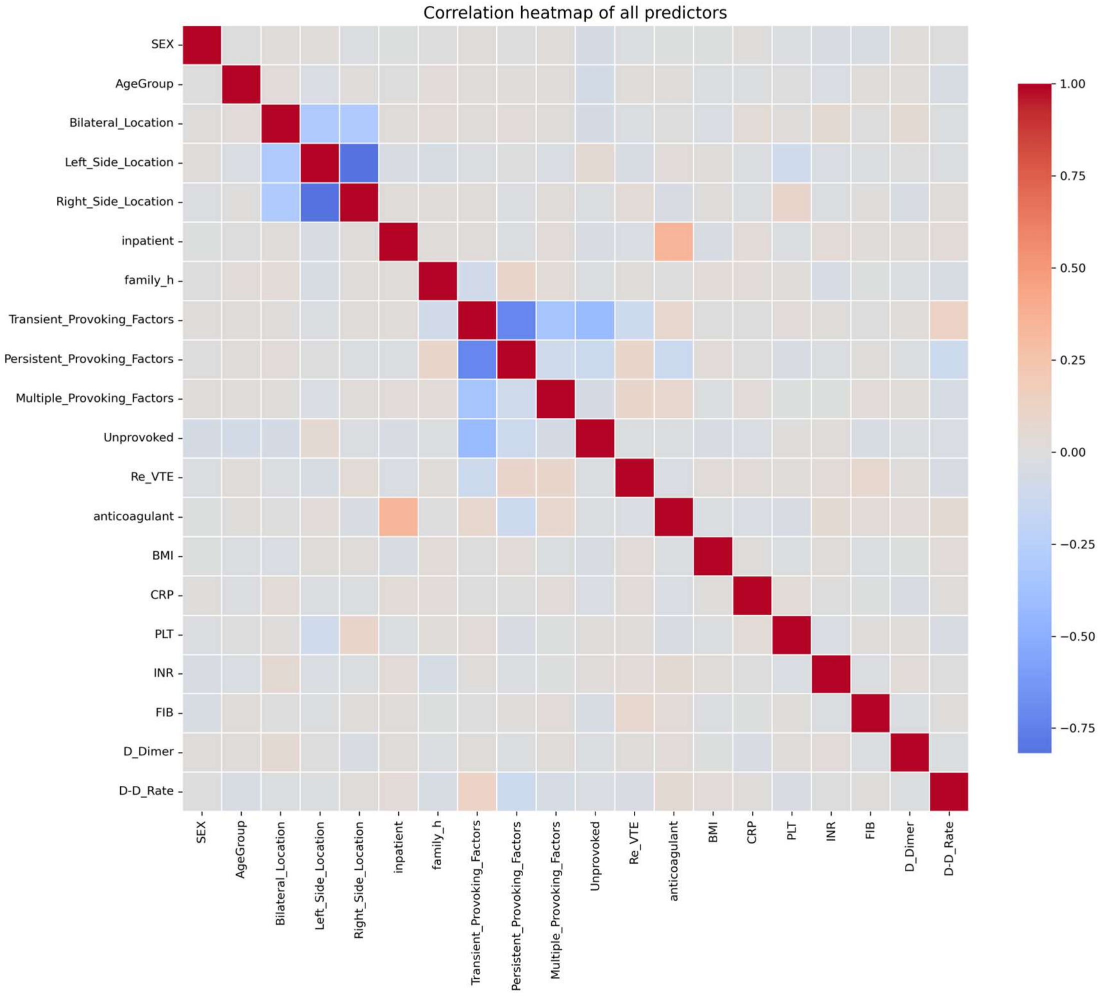

Correlation Heatmap of Predictors Included in the Logistic Regression Model:

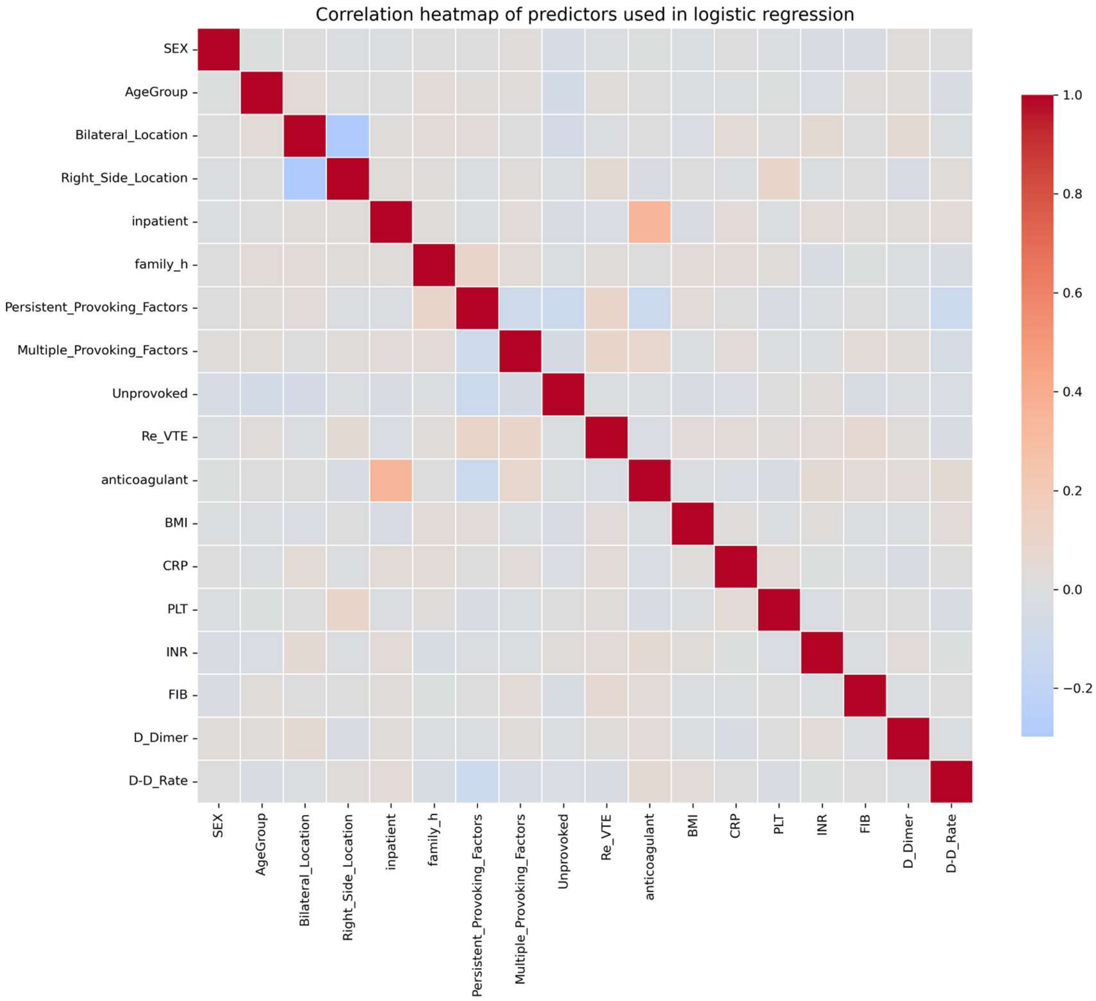

VIF Analysis of Predictors Included in the Logistic Regression Model:

**Variable**	**VIF**
anticoagulant	1.171718
inpatient	1.149025
Right_Side_Location	1.118837
Bilateral_Location	1.118410
Persistent_Provoking_Factors	1.079179
Unprovoked	1.039140
Multiple_Provoking_Factors	1.038066
Re_VTE	1.032377
D-D_Rate	1.026349
family_h	1.021027
PLT	1.017906
INR	1.015200
FIB	1.012612
CRP	1.012310
AgeGroup	1.011559
D_Dimer	1.009611
BMI	1.009465
SEX	1.007096
