# Supplementary material for: Machine learning-based model for predicting recanalization in isolated distal deep vein thrombosis and analysis of predictors
Source: PLoS One. 2026 May 8;21(5):e0349110. doi: 10.1371/journal.pone.0349110 (PMC13155594; doi:10.1371/journal.pone.0349110)
Supplement: S1 Table — (PDF) [file pone.0349110.s006.pdf]

Supplementary Table 1. Baseline characteristics of the development and test sets

Variable	Category	Overall	Development set	Test set	P value	Summary format	Test method
Sex	Male	796 (49.8%)	636 (49.7%)	160 (50.0%)	0.970	n (%)	Chi-square test
	Female	804 (50.2%)	644 (50.3%)	160 (50.0%)			
Age group	> 60 years	933 (58.3%)	749 (58.5%)	184 (57.5%)	0.790	n (%)	Chi-square test
	≤ 60 years	667 (41.7%)	531 (41.5%)	136 (42.5%)			
Thrombus location	Bilateral	160 (10.0%)	133 (10.4%)	27 (8.4%)	0.349	n (%)	Chi-square test
	Left side	729 (45.6%)	573 (44.8%)	156 (48.8%)			
	Right side	711 (44.4%)	574 (44.8%)	137 (42.8%)			
Hospitalization status	Inpatient	1037 (64.8%)	827 (64.6%)	210 (65.6%)	0.783	n (%)	Chi-square test
	Outpatient	563 (35.2%)	453 (35.4%)	110 (34.4%)			
Family history	Yes	73 (4.6%)	67 (5.2%)	6 (1.9%)	0.015	n (%)	Chi-square test
	No	1527 (95.4%)	1213 (94.8%)	314 (98.1%)			
Provoking factors	Transient provoking factors	1157 (72.3%)	927 (72.4%)	230 (71.9%)	0.158	n (%)	Chi-square test
	Persistent provoking factors	266 (16.6%)	218 (17.0%)	48 (15.0%)			
	Multiple provoking factors	74 (4.6%)	61 (4.8%)	13 (4.1%)			
	Unprovoked	103 (6.4%)	74 (5.8%)	29 (9.1%)			
VTE history	Recurrent VTE	261 (16.3%)	208 (16.2%)	53 (16.6%)	0.960	n (%)	Chi-square test
	First episode	1339 (83.7%)	1072 (83.8%)	267 (83.4%)			
Anticoagulant therapy	Yes	1127 (70.4%)	898 (70.2%)	229 (71.6%)	0.671	n (%)	Chi-square test
	No	473 (29.6%)	382 (29.8%)	91 (28.4%)			

Laboratory parameters	BMI	23.91 ± 3.26	23.89 ± 3.25	23.97 ± 3.30	0.705	mean ± SD	Welch's t-test
	CRP	5.06 (2.74, 7.30)	5.09 (2.78, 7.28)	4.94 (2.43, 7.31)	0.495	median (IQR)	Mann–Whitney U test
	Platelet count	235.00 (174.00, 292.00)	234.00 (173.00, 293.00)	237.50 (180.50, 289.75)	0.532	median (IQR)	Mann–Whitney U test
	INR	1.06 (0.97, 1.15)	1.06 (0.96, 1.15)	1.08 (0.98, 1.15)	0.079	median (IQR)	Mann–Whitney U test
	Fibrinogen	3.09 (2.53, 3.65)	3.11 (2.53, 3.65)	3.01 (2.52, 3.66)	0.381	median (IQR)	Mann–Whitney U test
	D-dimer	1.98 (1.25, 2.67)	2.00 (1.24, 2.70)	1.88 (1.29, 2.51)	0.206	median (IQR)	Mann–Whitney U test
	D-dimer reduction rate	0.25 (0.09, 0.42)	0.24 (0.09, 0.42)	0.29 (0.09, 0.43)	0.410	median (IQR)	Mann–Whitney U test
