# Supplementary material for: Machine learning-based model for predicting recanalization in isolated distal deep vein thrombosis and analysis of predictors
Source: PLoS One. 2026 May 8;21(5):e0349110. doi: 10.1371/journal.pone.0349110 (PMC13155594; doi:10.1371/journal.pone.0349110)
Supplement: S2 Table — (PDF) [file pone.0349110.s007.pdf]

| Abbreviation | Full term |
|---|---|
| AUC | Area under the receiver operating characteristic curve |
| BMI | Body mass index |
| CI | Confidence interval |
| CRP | C-reactive protein |
| DVT | Deep vein thrombosis |
| FIB | Fibrinogen |
| IDDVT | Isolated distal deep vein thrombosis |
| INR | International normalized ratio |
| LR | Logistic regression |
| ML | Machine learning |
| MLP | Multilayer perceptron |
| NPV | Negative predictive value |
| PDVT | Proximal deep vein thrombosis |
| PE | Pulmonary embolism |
| PLT | Platelet count |
| PPV | Positive predictive value |
| PTS | Post-thrombotic syndrome |
| RF | Random forest |
| ROC | Receiver operating characteristic |
| SHAP | SHapley Additive exPlanations |
| SVM | Support vector machine |
| VTE | Venous thromboembolism |
| XGBoost | Extreme gradient boosting |
